# A Sequential Inspection Procedure for Fault Detection in Multistage Manufacturing Processes

**DOI:** 10.3390/s21227524

**Published:** 2021-11-12

**Authors:** Rubén Moliner-Heredia, Gracia M. Bruscas-Bellido, José V. Abellán-Nebot, Ignacio Peñarrocha-Alós

**Affiliations:** Department of Industrial Systems Engineering and Design, Universitat Jaume I, Av. Vicent Sos Baynat, s/n, 12071 Castellón de la Plana, Spain; rmoliner@uji.es (R.M.-H.); bruscas@uji.es (G.M.B.-B.); ipenarro@uji.es (I.P.-A.)

**Keywords:** sequential inspection, fault detection, multistage process, information gain, Bayesian inference

## Abstract

Fault diagnosis in multistage manufacturing processes (MMPs) is a challenging task where most of the research presented in the literature considers a predefined inspection scheme to identify the sources of variation and make the process diagnosable. In this paper, a sequential inspection procedure to detect the process fault based on a sequential testing algorithm and a minimum monitoring system is proposed. After the monitoring system detects that the process is out of statistical control, the features to be inspected (end of line or in process measurements) are defined sequentially according to the expected information gain of each potential inspection measurement. A case study is analyzed to prove the benefits of this approach with respect to a predefined inspection scheme and a randomized sequential inspection considering both the use and non-use of fault probabilities from historical maintenance data.

## 1. Introduction

In the last several years, international institutions such as the European Factories of the Future Research Association (EFFRA) have promoted the development of strategies for modeling, monitoring, and controlling complex manufacturing systems to achieve zero-defects [1].

Multistage Manufacturing Processes (MMPs) are sequential manufacturing processes where workpieces move throughout different stages in order to perform specific manufacturing operations (e.g., welding, machining, etc.). Typical MMP in the industry are automotive body assemblies, machining lines, rolling processes, tile manufacturing processes, etc. One of the main characteristics of MMPs is the complex interactions among stages that define the final quality of the product. This is mainly due to the fact that the output quality at one stage is affected by the output quality of preceding stages. This complexity makes their control and quality assurance challenging.

If attention is focused on quality assurance in MMP, inspection allocation, monitoring, and fault diagnosis/identification are key issues that should be studied in detail. Many research works have been published on these topics in the last decade, and interesting surveys and reviews can be found in recent works [2,3,4,5]. 

In the field of fault diagnosis, a model that relates key product characteristics (KPCs) to sources of variation is needed for an effective root cause analysis. This model can be defined by engineering or data-driven approaches. A model based on engineering approaches can be obtained by deriving the physical laws that explain the process, e.g., kinematic relationships in assembly processes. A well-known engineering-based model in MMPs is the Stream of Variation (SoV) model [6] which has been successfully applied for fault diagnosis in different research. Zhou et al. [7] showed in detail the characteristics of the MMP for a fully diagnosable system considering the SoV model as a linear mixed-effects model. Conditions for the diagnosability property and the concept of minimal diagnosable class to analyze partial diagnosable systems were also illustrated. Ding et al. [8] compared different online variation estimators given continuous dimensional measurements for fault diagnosis purposes. In [9], the root-cause identification is formulated as a problem of estimation and hypothesis testing. In this work, online batch algorithms for the mean and variance estimation together with the hypothesis-testing methods for root-cause identification are illustrated. Sales-Setién et al. [10] proposed a recursive algorithm to estimate the process variance instead of online batch estimators, which reduces the computational cost and the data storage needs. Ding et al. [11] used the engineering model and the measurements at the inspection stage to identify fixture faults by a pattern recognition strategy based on principal component analysis. Although some fixtures presented the same pattern error on KPCs and, therefore, cannot be diagnosable, the fault patterns between stations were diagnosable. Xiang and Tsung [12] described how to define a control chart for statistical process control in an MMP based on the SoV model. The complex multi-stage monitoring problem is converted to a simple multi-stream monitoring problem by applying group exponential weighted moving average (EWMA) charts to the one-step ahead forecast errors of the model. The faulty stage is identified according to the results of the one-step ahead forecast errors. In a similar work, Li and Tsung [13] used the SoV model and EWMA charts for detecting and identifying the faults that affect the process covariance matrix in MMPs.

On the other hand, data-driven models are based on shop-floor data to extract the spatial pattern vectors (SPVs) that define the relationships between KPCs and sources of variation. Jin and Zhou [14] extracted the SPVs from the inspection data (sample covariance matrix), and they are compared with SPVs that have been previously extracted and whose sources of variation have been identified. Shan and Apley [15] proposed various blind source separation criteria to estimate the SPVs. Liu et al. [16] proposed the use of a qualitative model to relate KPCs with sources of variation instead of the SoV model and used this information to adjust in a proper way the SPVs extracted from data-driven approaches. The use of both engineering approaches (i.e., the qualitative model) and the data-driven approaches allow for explaining in a better way the extracted SPVs from the data.

Other advance modeling techniques such as Hierarchical Bayesian Networks (HBNs) have also been applied for monitoring and fault diagnosis in MMPs. In [17], an HBN is built using only data (process model is unknown) and once the network has been trained, the HBN is used to infer the unobserved inputs of the process (sources of variation). The identification of the fault and its type (mean shift or variance change) is accomplished by a control chart using the measured data and the inferred value from the HBN. Another HBN is proposed in [18] to deal with fault diagnosis in MMPs when the process is underdetermined. Under the assumption that less process faults are more likely to occur in MMPs, the problem of fault diagnosis is transformed into searching the sparse solution of abnormal variance changes for process faults. A similar problem is covered in [19], where the authors proposed a spatially correlated Bayesian learning algorithm for fault diagnosis. The algorithm is based on the relevance vector machine (RVM) exploiting the spatial correlation of dimensional variation from various process errors and a real automotive assembly is used to validate the effectiveness of the algorithm. Other artificial intelligence techniques have been explored for defect detection in similar contexts of MMPs and interesting reviews can be found in [20,21]. In [22], supervised and unsupervised learning approaches were explored to estimate healthy and unhealthy parts along the manufacturing process using different sensors data such as dynamometers, accelerometers, thermocouples, etc. Although this research does not deal with fault diagnosis, the estimation is used to reduce the number of inspections to be conducted since only those where the estimation cannot be ensured within a certain level of confidence are conducted. Beruvides et al. [23] presented a fault pattern identification methodology for multistage assembly processes with non-ideal sheet metal parts. Three different supervised and unsupervised neural network topologies (multi-layer perceptron network -MLP-, self-organized map -SOM-, and an MLP with genetic algorithms) with a Q-learning algorithm were implemented to compose a fault pattern identification library. All three methods were validated in a case study and the SOM network presented the best accuracy for fault pattern identification.

However, despite the large contributions in the field of fault diagnosis in MMPs, most of the research works are based on the existence of diagnosability conditions [7], which means that enough measurements are available to detect and identify the source of variation. Furthermore, these measurements are available at any time and almost at any station, since the diagnosability condition requires a large amount of data with enough information to isolate and identify the sources of variation. However, this approach may be not easy to be implemented in industry. Despite current trends of Industry 4.0, the cost of implementation and use at any time all measurements in a MMP may produce an important cost. Note that not only on machine measurements which could be non-invasive and without operator’s action are considered, but also in process measurements that may require use of CMM, gaging systems, etc. Therefore, a more conservative approach where the measurements are conducted only when the search of a root cause is necessary may be of great interest.

This paper proposes a sequential inspection procedure for fault diagnosis in MMP where, instead of measuring at any time most of the stages needed for full diagnosticability, the fault diagnosis is conducted in a sequential way. The proposed system is based on two parts. In the first part, a monitoring system is implemented to identify if the process is out of control. In the second part, a sequential inspection based on the evaluation of the information gain of each potential inspection measurement is conducted to detect the existing fault in the process. Note that the purpose of the system is to detect and isolate the fault, but there is no need for a complete identification of the fault, i.e., we want to know which fault exists without estimating its value. The methodology presented in this paper is based on a qualitative model of process faults and KPCs, which is derived using a type of tree diagram commonly applied in tolerance charting. This model is used instead of engineering models (e.g., SoV model) which can be difficult to derive for practitioners.

This paper is organized as follows. Section 2 shows the problem description and the proposed methodology for the sequential inspection procedure. Section 3 illustrates how to derive the qualitative model between sources of variation and KPCs using a graphical tree commonly applied in tolerance charting. Section 4 shows the minimum monitoring system that is needed to ensure all sources of variation can be detected. Section 5 presents the proposed sequential inspection methodology for a rapid inspection sequence and fault detection. Different case studies are analyzed under the proposed inspection approach and the results are compared with other possible inspection schemes in Section 6 and Section 7. Finally, Section 8 points out the main conclusions of the paper.

## 2. Problem Description

**Figure 1:**
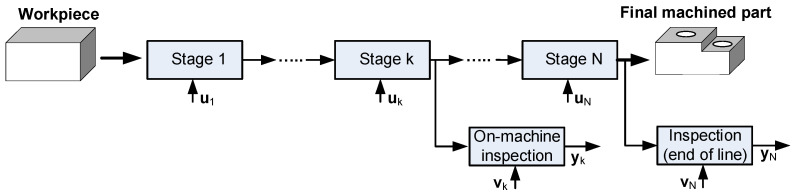
Multistage Manufacturing Process (MMP) with N stages. Notation: variations sources (**u**), measurement noise (**v**), inspection measurements (**y**).

Let us consider a MMP as shown in Figure 1, where the raw material starts at stage 1 and undergoes a series of manufacturing operations until the last stage, N. At each stage, critical process characteristics may affect the results on part quality, for instance, a fixture locator which plays a critical role in determining the dimensional quality of an assembled or machined part. These critical characteristics are called key control characteristics (KCC), and their deviations from their nominal values at stage *k* are denoted as **u**_k_. The quality of the part is evaluated through an inspection stage or by on machine measurements and the deviations of KPCs from nominal values at stage *k* are denoted by **y**_k_. If a linear model links the deviations of KCCs (i.e., sources of variation) with the deviations of KPCs derived from measurements, the following equation is defined:

(1) y=Γ·u+ε,

where $y={\left[y_{1}^{T}\;y_{2}^{T}\;\ldots \;y_{N}^{T}\right]}^{T}$ is an m×1 vector that represents the measured dimensional deviation of KPCs from station 1 to station N; $u={\left[u_{1}^{T}\;u_{2}^{T}\;\ldots \;u_{N}^{T}\right]}^{T}$ is an n×1 vector that represents the deviations of KCCs up to station N; Γ is the fault pattern matrix (m×n) that can be derived from engineering or data-driven approaches; and ε denotes a term that includes both the modeling uncertainty and the measurement noise (**v**).

As shown in Equation (1), to fully identify all sources of variation, measurements along the MMP should be conducted. The diagnosability of these sources of variation and the final inspection cost are the main issues in the design of the inspection scheme in MMPs for fault diagnosis and quality assurance.

Given this MMP, the following questions may arise: which KPCs should be inspected for monitoring the process at the end of line? Which stages/KPCs should be inspected for fault diagnosis purposes? Which inspection sequence should be followed to identify the sources of variation with a minimum number of measurements? Note that previous research has dealt with similar problems, but, after the definition of the inspections stations, the measurements were assumed to be obtained at any time. In the presented problem, a sequential approach is proposed and thus the decision of which stage or KPC should be inspected depends on the results of previous inspections.

To solve this problem, the following 3-steps methodology is proposed:

1. Derivation of a qualitative model between sources of variation and KPCs.
2. Definition of a minimum monitoring system to trigger the sequential inspection procedure.
3. Sequential inspection procedure based on the Information Gain (IG).

For the research in this paper, the following is assumed:

- The analyzed MMP is composed of stations that conduct machining operations, and, therefore, the potential process faults are related to fixtures and cutting tools.
- Only one fault exists at the same time in the MMP.
- Type I errors (true conforming parts are considered nonconforming after inspection) and Type II errors (true nonconforming parts are considered conforming after inspection) are assumed to be negligible.

**Figure 2:**
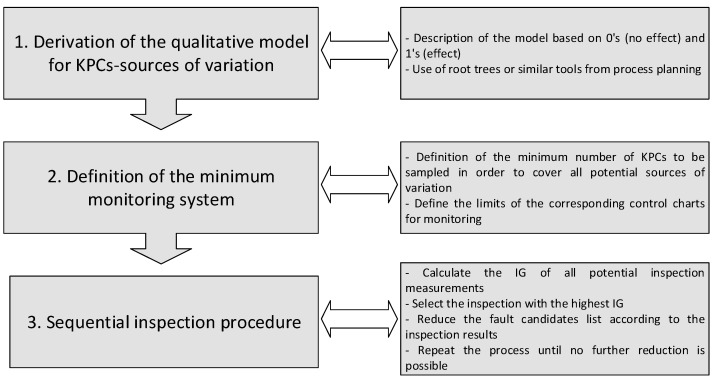
Methodology overview for fault detection based on sequential inspection.

The following sections show in detail the three-step methodology proposed, which is illustrated in Figure 2.

## 3. Qualitative Model of KPCs-Process Faults

The qualitative model of KPCs-process faults refers to the qualitative estimation of matrix Γ from Equation (1). As explained above, this matrix can be obtained from engineering or data-driven approaches. However, in this paper, the use of a simpler model considering the qualitative relationships of the MMP to indicate which source of variation influences on which KPCs is explored. If a relationship exists, the corresponding Γ coefficient has a value of 1. Otherwise, the value is 0.

The qualitative model is extracted from the process planning information, more specifically from tolerance charting. Tolerance charting is a common activity that is performed in process planning to ensure that design tolerances can be achieved. To analyze the variation propagation and estimate if the part is within specifications, a root tree and a tolerance chart are built. The root tree is a graphical representation of the process where the sequence of machined surfaces and datums (surfaces used for locating the workpiece in the fixture) can be extracted. A brief explanation of the rooted tree is given in [24].

In this paper, the following modification of the rooted tree for deriving a qualitative model of KPCs-process faults is proposed:

- Machining operations that are conducted with the same tool are represented with the same type of arrow at each subjob/stage.
- If a feature previously machined is used as datum downstream, the feature is drawn two times connected by a thick line.
- Whether on-machine measurement inspections are conducted and the potential process faults are indicated on the right-hand side of the rooted tree. Two types of process faults are distinguished: (i) cutting tool faults (excessive wear or breakage), denoted as **u_f_**; (ii) fixture faults (deviations of locators or workholding devices), denoted as **u_m_**. Similarly, two types of on-machine inspections are distinguished: (i) tool inspection or KPC inspection, denoted as **y_um_**; (ii) fixture inspection, denoted as **y_uf_**.

Furthermore, it is assumed that, for the purpose of fault detection, the machining error due to machine-tool precision is negligible, and thus the machining error only refers to cutting tool errors due to excessive tool wear or tool breakage.

**Figure 3:**
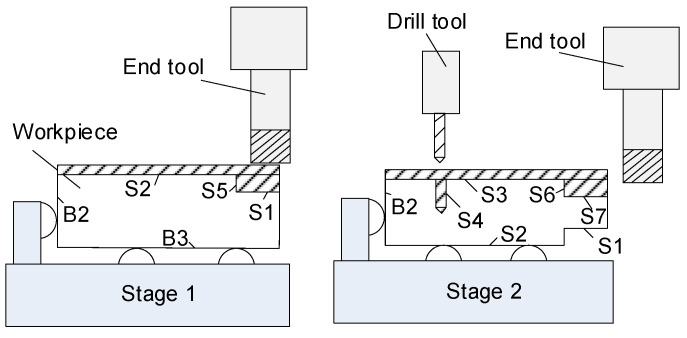
Example of an MMP to illustrate the qualitative model.

**Figure 4:**
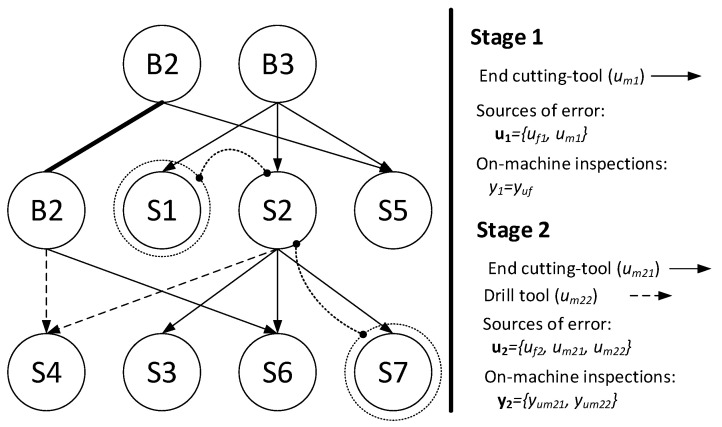
Resulting rooted tree of a previous MMP example. The path related to KPC1 (distance between S1 and S7) is shown in dotted lines to clarify the methodology to obtain the matrix Γ in the text. B2–B3, raw surfaces; S1–S7, machined surfaces.

To illustrate the rooted tree for a MMP with the above modifications, let us consider the MMP shown in Figure 3. The process plan is as follows. At stage 1, the workpiece is clamped using as datum the raw surfaces B2 and B3, and it is machined with the same cutting tool to obtain surfaces S1 and S2. At stage 2, the workpiece is located using the datum surfaces S2 and B2. At this stage, surfaces S6 and S7 are machined with the same end mill tool; surface S4 is generated using a drilling tool. The KPCs that are of interest according to the drawing specifications are: KPC1, distance between S7 and S1; KPC2, distance between S3 and S2; KPC3, distance between S6 and S4. Under this process plan, the resulting rooted tree is shown in Figure 4.

Given the information from the rooted tree and the KPCs, the derivation of matrix Γ that connects the sources of variation with the KPCs can be easily obtained. The matrix is drawn following the procedure shown below:

- Look for the features that define the KPCs. For instance, KPC1 is the distance between S7 and S1.
- Find the path that connects both features.
- Each path defines the row of matrix Γ. This row is defined by 1′s or 0′s as follows:
  ○ An arrow means a cutting tool error, thus 1 is set to the corresponding column of this cutting tool error.
  ○ When the path moves from one stage to the next one, a fixture error is added from the first stage; thus, 1 is set to that fixture error.
  ○ If a path includes a thick line, this line does not add any value in the model.
  ○ If the path includes two machined features in the same stage, no fixture error is added (the fixture errors are compensated); thus, a 0 is set to that fixture error. Similarly, if the cutting tool is used to machine both surfaces, a 0 is also set in the corresponding cutting tool error. If different cutting tools are used, a 1 is set to each corresponding cutting tool error.
  ○ Any error that is not identified in the path is set to 0.
  ○ For on-machine measurements of fixtures, set 1 to those fixture errors.
  ○ For on-machine measurements of cutting tools (surface inspections with a touch probe on machine or direct inspection of tools), a 1 is set to cutting tool errors at that stage.

To illustrate the procedure, let us consider the KPC1 which is defined by the distance between surface S7 and surface S1. The path that connects both surfaces is illustrated in Figure 4 using dotted lines. As it can be seen, from S7 to S2, there is an arrow that represents the machining process with the end cutting tool, so this source of error is set to 1 (*u_m_*_21_). Then, surface 2 is used as datum and thus the fixture error of stage 2 is added (*u_f_*_2_). Finally, the path moves from surface 2 to surface 1 using the datum B3. Both surfaces are machined with the same tool and same datum, so no additional errors are added. Therefore, the row of Γ matrix for the KPC1 is [0, 0, 1, 1, 0]. Note that the source of errors is **u** = [*u_f_*_1_*, u_m_*_1_*, u_f_*_2_*, u_m_*_21_*, u_m_*_22_]*^T^*.

As a result of applying this procedure, the qualitative model KPCs-sources of variation is defined as:

(2) $$y=\left[\begin{array}{c} y_{\mathrm{on}-\mathrm{machine}}\\ y_{\mathrm{end}-\mathrm{line}} \end{array}\right]=\Gamma \cdot u=\left[\begin{array}{c} \Gamma_{\mathrm{on}-\mathrm{machine}\;}\\ \Gamma_{\mathrm{end}-\mathrm{line}\;} \end{array}\right]\cdot \left[\begin{array}{c} u_{f1}\\ u_{m1}\\ \begin{array}{c} u_{f2}\\ u_{m21}\\ u_{m22} \end{array} \end{array}\right],\;$$

(3) $$\left[y_{\mathrm{on}-\mathrm{machine}}\right]=\left[\begin{array}{c} y_{uf1}\\ y_{um2} \end{array}\right]=\Gamma_{\mathrm{on}-\mathrm{machine}\;}\cdot \left[\begin{array}{c} u_{f1}\\ u_{m1}\\ \begin{array}{c} u_{f2}\\ u_{m21}\\ u_{m22} \end{array} \end{array}\right]=\left[\begin{array}{ccc} 1 & 0 & \begin{array}{cc} 0 & \begin{array}{cc} 0 & 0 \end{array} \end{array}\\ 0 & 0 & \begin{array}{cc} 0 & \begin{array}{cc} 1 & 1 \end{array} \end{array} \end{array}\right]\cdot \left[\begin{array}{c} u_{f1}\\ u_{m1}\\ \begin{array}{c} u_{f2}\\ u_{m21}\\ u_{m22} \end{array} \end{array}\right],\;$$

(4) $$\left[y_{\mathrm{end}-\mathrm{line}}\right]=\left[\begin{array}{c} KPC_{1}\\ KPC_{2}\\ KPC_{3} \end{array}\right]=\Gamma_{\mathrm{end}-\mathrm{line}\;}\cdot \left[\begin{array}{c} u_{f1}\\ u_{m1}\\ \begin{array}{c} u_{f2}\\ u_{m21}\\ u_{m22} \end{array} \end{array}\right]=\left[\begin{array}{c} \begin{array}{ccc} 0 & 0 & \begin{array}{cc} 1 & \begin{array}{cc} 1 & 0 \end{array} \end{array}\\ 0 & 0 & \begin{array}{cc} 1 & \begin{array}{cc} 1 & 0 \end{array} \end{array} \end{array}\\ \begin{array}{ccc} 0 & 0 & \begin{array}{cc} 0 & \begin{array}{cc} 1 & 1 \end{array} \end{array} \end{array} \end{array}\right]\cdot \left[\begin{array}{c} u_{f1}\\ u_{m1}\\ \begin{array}{c} u_{f2}\\ u_{m21}\\ u_{m22} \end{array} \end{array}\right].\;$$

Therefore, the Γ matrix is

(5) $$\Gamma =\left[\begin{array}{c} \begin{array}{ccc} 1 & 0 & \begin{array}{cc} 0 & \begin{array}{cc} 0 & 0 \end{array} \end{array}\\ 0 & 0 & \begin{array}{cc} 0 & \begin{array}{cc} 1 & 1 \end{array} \end{array} \end{array}\\ \begin{array}{c} \begin{array}{ccc} 0 & 0 & \begin{array}{cc} 1 & \begin{array}{cc} 1 & 0 \end{array} \end{array}\\ 0 & 0 & \begin{array}{cc} 1 & \begin{array}{cc} 1 & 0 \end{array} \end{array} \end{array}\\ \begin{array}{ccc} 0 & 0 & \begin{array}{cc} 0 & \begin{array}{cc} 1 & 1 \end{array} \end{array} \end{array} \end{array} \end{array}\right],\;$$

## 4. Definition of the Monitoring System

The purpose of the sequential inspection approach is to conduct the search for the root causes only when the process is detected to be statistically out of control. Up to this moment, only a minimum number of KPCs should be inspected, reducing the inspection costs. Therefore, it is important to define the minimum KPCs to be inspected in order to be sensitive to all sources of variation. In some MMPs, due to variation propagation, only the inspection of some KPCs at the end of line may be enough to have a good indicator about the general state of the process. If these KPCs are within statistical control, it can be assumed that all sources of variation are under admissible levels and no further inspections are required. 

**Figure 5:**
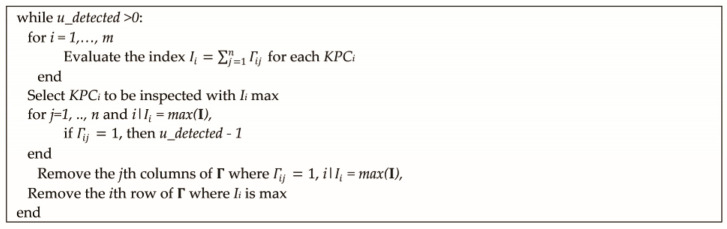
Algorithm to define the minimum KPCs to be inspected for indirectly monitoring all sources of variation.

Given the qualitative model previously defined, the minimum monitoring system that includes the effects of all sources of variation can be derived through a basic search algorithm. Figure 5 shows the proposed search algorithm to identify the minimum KPCs that are required to be monitored.

Given the set of KPCs to be inspected at the end of the line, a quality control system based on control charts can be built to monitor the state of the process. After setting the control limits of the control chart for each KPC, the monitoring system can be used to detect if the process is out of statistical control. See [25] for more details of setting control chart limits. At that moment, the sequential inspection procedure, derived in the following section, can be executed to detect the existing fault process. 

## 5. Sequential Inspection Methodology

The sequential inspection methodology is based on the evaluation of the information gain every time an inspection is conducted, and the source of variation has not been identified yet. The proposed methodology is based on a sequential approach that has been successfully applied in the field of software testing [26,27].

### 5.1. Bayesian Approach for Diagnostic Explanation

The sequential inspection approach defines which sequential measurements along the process should be conducted based on the fault probabilities estimated by the Bayesian reasoning, which is updated after an inspection measurement is carried out. 

The starting point is a set of diagnostic explanations that indicate which fault process may exist in the system, denoted as **D** = *{d*_1_*, …, d_n_}*. Since it is assumed that only one fault is active at the same time, d_k_ refers to a specific process fault u_k_ that is present in the system; thus, **D** = *{u*_1_*, …, u_n_}*. The finite set of inspection measurements is defined as **Y** = *{y*_1_*, …, y_m_}*, and the result of the inspection can be 0 (inspected feature is within statistical control) or 1 (the feature inspected is out of control). The result of the *y_i_* inspection is defined as *o_i_*, and *o_i_* = 0 or 1. The prior probability of the process fault is obtained according to maintenance data or, if does not exist, an equal probability of all faults is given. 

According to previous nomenclature, the prior probability of a diagnostic explanation where *u_k_* is faulty is

(6) $$Pr\left(d_{k}\right)=Pr\left(u_{k}\right)=\frac{1}{n},$$

if no maintenance data are applied. 

In order to apply the sequential inspection procedure, the probability of this diagnostic explanation needs to be estimated if the inspection result from *y_i_* (i.e., $o_{i}$) is that the feature is out of control. Therefore, according to Bayes’ rule,

(7) $$Pr\left(d_{k}|o_{i}=1\right)=\frac{Pr(o_{i}=1|d_{k})}{Pr\left(o_{i}\right)}\cdot Pr\left(d_{k}\right).$$

In this equation, $Pr(o_{i}=1|d_{k})$ represents the probability of the observed outcome, if that diagnostic explanation *d_k_* is the correct one, given by

(8) $$Pr\left(o_{i}=1|d_{k}\right)=1-Pr\left(o_{i}=0|d_{k}\right)=\Gamma_{ik}.$$

Note that, according to the qualitative model, if *u_k_* is faulty, the *i*th inspection measurement will be out of control if $\Gamma_{ik}=1$. The term $Pr\left(o_{i}\right)$ represents the probability of the observed outcome, independently of which diagnostic explanation is the correct one. The value of $Pr\left(o_{i}\right)$ is a normalizing factor that is given by

(9) $$Pr\left(o_{i}\right)=\underset{d_{k}\in D}{\sum }Pr\left(o_{i}|d_{k}\right)\cdot Pr\left(d_{k}\right).\;$$

### 5.2. Priorization Based Information Gain

The priorization of the inspection measurement is based on maximizing the Information Gain (IG) index defined by Johnson [28]. The IG is defined as

(10) $$\mathrm{IG}\left(D,y_{i}\right)=H\left(D\right)-Pr\left(o_{i}=0\right)\cdot H\left(D_{0}\right)-Pr\left(o_{i}=1\right)\cdot H\left(D_{1}\right),$$

where **D_0_** and **D_1_** represent the updated diagnosis explanation if inspection *y_i_* results in a feature within control or out of control, respectively. The entropy of a set of diagnostic candidates **D**, denoted as *H(***D***)*, is defined as

(11) $$H\left(D\right)=-\sum_{d_{k}\in D}Pr\left(d_{k}\right)\cdot \log_{2}\left(\mathrm{Pr}\left(d_{k}\right)\right),$$

which can be understood as the average information we are missing until we can be certain about the diagnosis [26,27]. Therefore, IG diagnostic prioritization integrates Bayesian diagnosis in the inspection sequence selection and uses the information gain as the main indicator to express the diagnostic utility of executing a specific inspection measurement. From a detection point of view, the best inspection to be conducted is the one that yields the highest IG.

**Figure 6:**
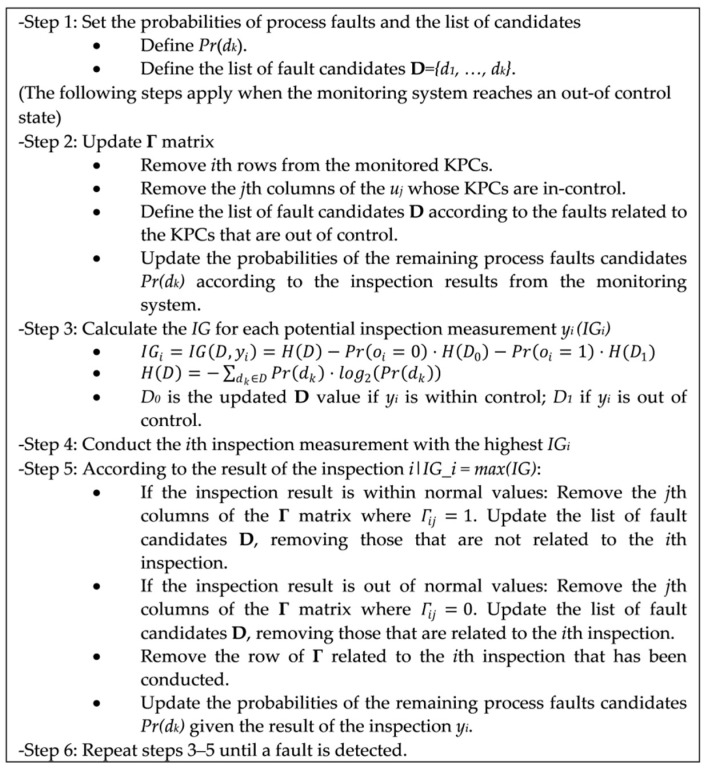
Algorithm for the sequential inspection procedure.

The algorithm to be implemented for the sequential inspection procedure is shown in Figure 6.

### 5.3. Effectiveness of the IG Approach

In order to analyze the effectiveness of the sequential inspection approach based on the IG versus an inspection approach based on random selection, let us consider a process with *n* sources of variation, and denote ρ as the coverage density that indicates the coverage of each inspection with respect to the sources of variation, i.e., the inspection is related to ρ·n sources of variation. This coverage factor is applied throughout all the sequential process, thus each inspection will be able to detect $\rho \cdot n_{r}$ sources of variation, where $n_{r}$ is the remaining sources of variation that have not been discarded yet.

According to [27], the IG index for a Γ matrix with a coverage density ρ is defined as

(12) $$\mathrm{IG}\left(\rho \right)=-\rho \cdot \log_{2}\left(\rho \right)-\left(1-\rho \right)\cdot \log_{2}\left((1-\rho \right)).$$

At this point, two extreme cases can be studied to analyze the effectiveness of the IG approach. First, the best case scenario corresponds to a sequential inspection scheme where the sources of variation are split in two equal sets of fault candidates, i.e., when ρ=0.5. Under this scenario, the IG index is maximum (IG = 1) and the average number of inspections required to detect the final fault is defined as $\log_{2}\left(n\right)$.

Secondly, the worst case scenario is when the inspections only detect the effect of one single fault. This case is given when the coverage density is ρ=1/n and thus the IG is minimum. Under this scenario, the average number of inspections required is $\frac{n+1}{2}-\frac{1}{n}$. It can be noted that, in this worst case scenario, there is no benefit of using the IG index since all potential inspections present a minimum value of IG, and the resulting sequential inspection is equal to a random sequential inspection approach.

**Figure 7:**
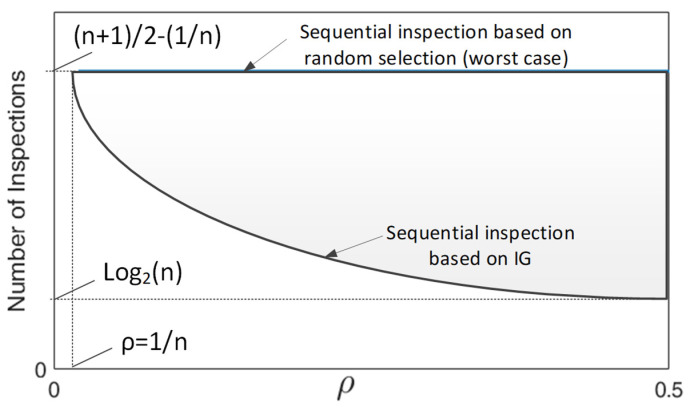
Expected evolution of the required average number of inspections for fault diagnosis under a sequential inspection based on IG and a random selection.

Figure 7 shows the expected evolution of the required number of inspections for a given coverage density  ρ under the sequential inspection approach based on the IG and based on a random selection. As it can be seen, the effectiveness of the IG approach increases when the coverage density increases. It is worth mentioning that MMP with a higher error propagation between stages present higher coverage densities and therefore the IG index may have an important impact on sequential inspection approaches. Please note that, in Figure 7, the random selection curve refers to the worst case within the random selection approach considering that, besides the inspections according to the given ρ, additional inspections to check single faults are available. Therefore, the real average number of inspections required under the random selection for a given MMP is expected to be between this worst case curve and the IG curve, and it will depend on the specific structure of the Γ matrix. 

## 6. Case Study

**Figure 8:**
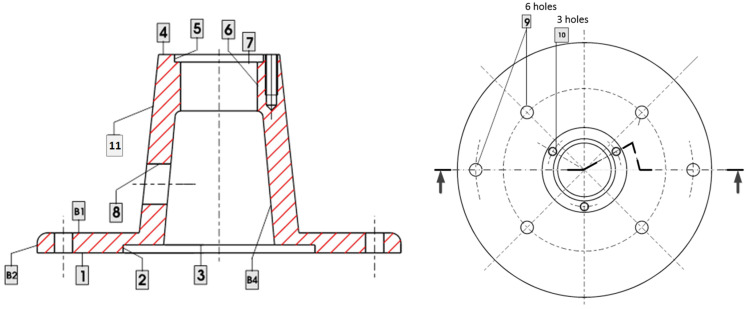
Part to be machined with numbered surfaces. B1, …, means raw surfaces. 1, 2, …, means machined surfaces and are referred in the text as S1, S2, etc.

To illustrate the performance of this sequential inspection methodology for fault detection, let us consider the part shown in Figure 8 that is manufactured according to the process plan presented in Table 1; Table 2 and Table 3 show the KPCs to be inspected and the on-machine measurements that can be conducted in the process. To evaluate the resulting cost of the inspection scheme, the inspection from KPC1 to KPC7 is set to 100 €, and the inspection from KPC8 to KPC13 is set to 115 €. The costs for on-machine inspections are set to 85 €. 

**Figure 9:**
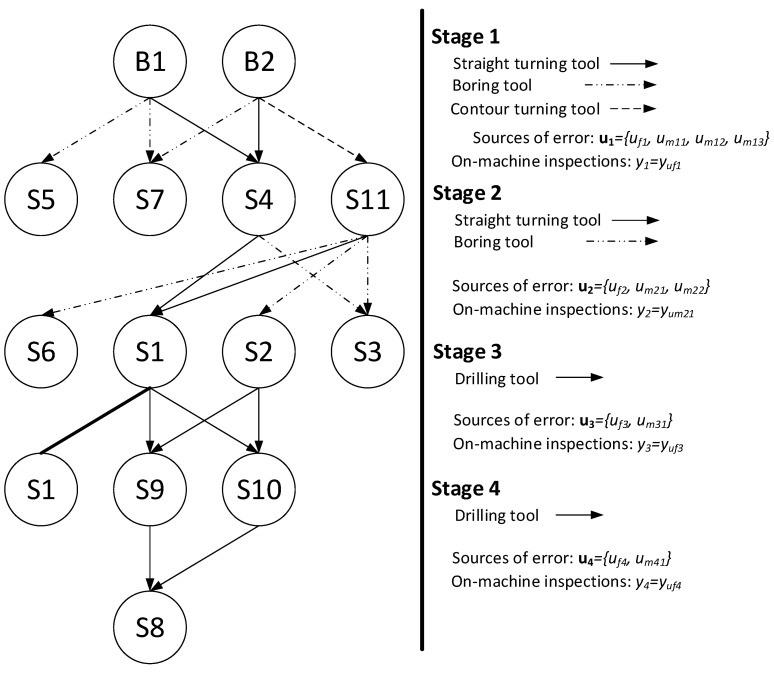
Rooted tree of the case study. B1–B2, raw surfaces; S1–S11, machined surfaces.

From the above process plan, the rooted tree shown in Figure 9 can be derived. As it can be seen, there are 11 potential process faults, 13 potential inspection measurements, and 4 on-machine measurements. From the rooted tree, the qualitative model that links process faults and inspection measurements is:

(13) $$y_{\mathrm{on}-\mathrm{machine}}=\left[\begin{array}{c} y_{uf1}\\ y_{um21}\\ \begin{array}{c} y_{uf3}\\ y_{uf4} \end{array} \end{array}\right]=\Gamma_{\mathrm{on}-\mathrm{machine}\;}\cdot u,\;$$

(14) $$y_{\mathrm{end}-\mathrm{line}}=\left[\begin{array}{c} y_{uf1}\\ y_{um21}\\ \begin{array}{c} y_{uf3}\\ y_{uf4} \end{array} \end{array}\right]=\Gamma_{\mathrm{end}-\mathrm{line}\;}\cdot u,\;$$

(15) $$y_{\mathrm{inspection}}=\left[\begin{array}{c} y_{\mathrm{on}-\mathrm{machine}}\\ y_{\mathrm{end}-\mathrm{line}} \end{array}\right]=\left[\begin{array}{c} \Gamma_{\mathrm{on}-\mathrm{machine}\;}\\ \Gamma_{\mathrm{end}-\mathrm{line}\;} \end{array}\right]\cdot u=\Gamma \cdot u,\;$$

where

(16) $$\Gamma_{\mathrm{on}-\mathrm{machine}\;}=\left[\begin{array}{ccccccccccc} 1 & 0 & 0 & 0 & 0 & 0 & 0 & 0 & 0 & 0 & 0\\ 0 & 0 & 0 & 0 & 0 & 1 & 0 & 0 & 0 & 0 & 0\\ 0 & 0 & 0 & 0 & 0 & 0 & 0 & 1 & 0 & 0 & 0\\ 0 & 0 & 0 & 0 & 0 & 0 & 0 & 0 & 0 & 1 & 0 \end{array}\right],$$

(17) $$\Gamma_{\mathrm{end}-\mathrm{line}\;}=[\begin{array}{ccccccccccc} 0 & 0 & 0 & 0 & 1 & 1 & 0 & 0 & 0 & 0 & 0\\ 1 & 0 & 1 & 1 & 1 & 1 & 1 & 1 & 1 & 1 & 1\\ 0 & 1 & 1 & 1 & 1 & 1 & 0 & 0 & 0 & 0 & 0\\ 0 & 1 & 1 & 1 & 1 & 1 & 1 & 1 & 1 & 0 & 0\\ 0 & 0 & 0 & 0 & 0 & 0 & 0 & 0 & 0 & 1 & 1\\ 0 & 1 & 0 & 1 & 1 & 0 & 1 & 0 & 0 & 0 & 0\\ 1 & 0 & 0 & 0 & 1 & 1 & 1 & 1 & 1 & 1 & 1\\ 1 & 0 & 1 & 1 & 1 & 1 & 0 & 0 & 0 & 0 & 0\\ 0 & 0 & 0 & 0 & 0 & 0 & 0 & 1 & 1 & 0 & 0\\ 0 & 1 & 0 & 1 & 0 & 0 & 0 & 0 & 0 & 0 & 0\\ 0 & 0 & 0 & 0 & 0 & 0 & 0 & 0 & 0 & 0 & 1\\ 0 & 0 & 0 & 0 & 0 & 0 & 1 & 0 & 0 & 0 & 0\\ 0 & 0 & 0 & 0 & 0 & 0 & 0 & 0 & 1 & 0 & 0 \end{array}].$$

According to Section 4, the KPCs that should be monitored to include all sources of variation are KPC2 and KPC3.

### Fault Detection Results and Discussion

In order to compare the performance of the proposed sequential inspection procedure, the number of inspections required to successfully detect the process faults under three different inspection schemes are compared:

(1) a full inspection system in order to make the process faults fully diagnosable. In this case, there is no sequential inspection since the minimum number of KPCs to be inspected is always measured for fault detection. For this case study, to fully detect any process fault, the following inspections are required: KPC4, KPC6, KPC7, KPC8, and on-machine inspections in stages 3 and 4. Therefore, a total of 6 inspections are needed. Note that any of the eleven potential faults can be fully detected and isolated by the combination of these six inspection measurements since the pattern fault defined by any of these 11 potential faults are different from each other.
(2) a random sequential inspection procedure. In this case, the proposed sequential inspection process is applied, but, instead of using the IG index, the KPCs to be inspected are randomly selected and the inspection result is used to reduce the potential process faults candidates of the system.
(3) the proposed sequential inspection procedure, where the required inspection measurements are selected according to the IG index.

The comparison is conducted in terms of both costs and number of KPCs to be inspected before a fault detection is reached. For the first scheme (fully diagnosable system), the number of the KPCs needed is 6 as stated above. For the other two schemes, Monte Carlo simulations are evaluated where, at each simulation, a fault is added into the system and the sequential procedure is launched in order to finally detect it. The average number of inspections needed after 11,000 simulations is considered as the performance value for comparison purposes. Additionally, two situations are analyzed: a first situation where there is no information about the prior fault probability; thus, equal fault probabilities are assumed; a second situation where the information from maintenance data is used and then the ratios of fault probabilities are known.

The results of the three schemes and the two situations are shown in Table 4. As it can be seen, the use of a sequential inspection procedure can reduce the number of inspection measurements needed with respect to a predefined inspection scheme. The fixed scheme requires a continuous inspection of six KPCs, whereas the sequential inspection reduces the average number of measurements needed to less than 5, which means more than 15% of reduction. Furthermore, the use of a random search in the sequential approach can sometimes give a smaller number of inspections required, but, taking into account the average from 11,000 simulations, the random approach requires more measurements than the sequential approach, 4.9 versus 4.2. Additionally, if the probabilities of process faults are known in advance, the IG algorithm can reach an average number of measurements of 4.0, slightly better than 4.2 that was obtained using equal probabilities of all process faults. Note that, for this case study, the number of process faults is not too large (only 11 faults), and the use of the prior probabilities from maintenance for a faster fault detection may not have a high impact. Comparing the predefined scheme with the sequential IG algorithm, the reduction of the number of inspections is from 6 to 4, which means a reduction of 33%. In terms of cost, the sequential approach based on the IG index can reduce the cost of inspection from 585 € to 389 € which means a similar percentage of cost reduction.

## 7. Additional Case Studies for Validation

The previous case study has shown the benefit of applying the proposed sequential approach based on IG for fault diagnosis in a 4-stage machining process. However, as it was pointed out in Section 5, the effectiveness of the methodology depends on the structure of the Γ matrix, i.e., it depends on the coverage density ρ. To have a more complete validation of the proposed methodology, two different scenarios are evaluated with a random generation of Γ matrices. 

For both scenarios, the number of sources of variation is set to *n* = 18, and the number of potential inspections is set to *m* = 30. For the first scenario, Γ matrix is randomly generated forcing 4/5 of the inspections to present a ρ of 0.5, and 1/5 of the inspections present a ρ of 0.1. This Γ matrix is considered a high-density matrix which would be the result of an MMP with a high error propagation via datums. The second scenario presents a Γ matrix randomly generated where 4/5 of the inspections present a ρ of 0.1, and 1/5 of the inspections a ρ of 0.5. This is an opposite scenario where a low error propagation exists along the MMP. Both scenarios are compared in terms of number of inspections required and total cost of the inspection scheme for fault diagnosis. The cost of each inspection is randomly set to 100 ± 20 € using a uniform distribution. All sources of variation have the same a priori probability of fail.

As it is shown in Table 5, the results validate the proposed methodology since the reduction of number of inspections and cost is relevant. However, as it was pointed out in Section 5, the benefit of the methodology increases when the Γ matrix presents a higher coverage density. In this case study, the reduction of number of inspections for the first scenario (a process with high error propagation and therefore higher ρ values) is 55% (from 1158 € to 521 €), whereas the reduction in the second scenario (a process with less error propagation and therefore lower ρ values) is only 13% (from 512 € to 445 €). 

## 8. Conclusions

Sequential inspection in MMPs can be of interest to reduce the inspection cost and provide fast fault detection. This paper has proposed a methodology to implement a sequential inspection procedure based on the information gain index of the inspection measurement. To evaluate this index, a qualitative model that links the sources of variation with the KPCs is derived. The methodology is composed of three parts: a qualitative model extracted from process planning; a monitoring system to detect if the process is out of statistical control; and a sequential inspection procedure applied for a fault detection search which is based on maximizing the information that can be obtained from a specific set of inspection measurements. 

The proposed methodology has been theoretically analyzed showing that the IG algorithm can highly reduce the number of inspections required when the MMP presents a high error propagation behavior, which means that the coverage density of the inspections tends to be high. Otherwise, when the MMP presents low error propagation and the coverage density of the inspection is low, the benefit of maximizing the IG instead of a random selection is lower. A more specific MMP based on four machining stages was also analyzed to prove the generation of the quality model through the graphical representation of the MMP and the reduction of the inspections required when a sequential inspection approach based on IG is applied. In this case study, a reduction of 33% in the inspection effort and cost was obtained with respect to common practices where a predefined inspection scheme for fault detection is given.

## Figures and Tables

**Table 1:** Manufacturing process plan for the case study.

	Stage 1	Stage 2	Stage 3	Stage 4
Machine-tool	Lathe	Lathe	Machining center	Machining center
Datum surfaces	B1, B2	S4, S11	S1, S2	S1, S9
Workholding system	3-jaw chuck & positioners	3-jaw chuck & positioners	3-jaw chuck & positioners	3 locators and concentric and radial locators
Machined features	S5, S7, S4, S11	S6, S2, S3, S1	S9, S10	S8

**Table 2:** KPCs for the part analyzed in the case study.

KPCs	Characteristic	KPCs	Characteristic	KPCs	Characteristic
KPC1	Distance S1–S4	KPC5	Distance S8–S1	KPC9	Position S9–S2
KPC2	Distance B1–S8	KPC6	Concentricity S2–S5	KPC10	Concentricity S11–S5
KPC3	Distance S7–S1	KPC7	Distance S4–S8	KPC11	Diameter S8
KPC4	Distance S10–S1	KPC8	Distance B1–S1	KPC12	Diameter S6
				KPC13	Diameter S9

**Table 3:** Possible on-machine measurements.

On-Machine Inspection	Characteristic
$y_{uf1}$	Fixture inspection stage 1
$y_{um21}$	Tool inspection stage 2
$y_{uf3}$	Fixture inspection stage 3
$y_{uf4}$	Fixture inspection stage 4

**Table 4:** Number of inspection measurements required for fault identification and cost. Three schemes: fully diagnosable, proposed sequential inspection with random selection of inspections, and proposed sequential inspection with IG index. Two situations: all fault probabilities are equal; fault probabilities are defined according to maintenance data.

Prior Fault Probabilities	Fully Diagnosable	Sequential Inspection but Random Selection	Sequential Inspection with IG Index
Equal probable *	6 (585 €)	5.0 (508 €)	4.2 (404 €)
Based on maintenance data **	6 (585 €)	5.0 (508 €)	4.0 (389 €)

Note that prior fault probabilities are only used in the proposed sequential algorithm. * All process faults have random probabilities, and these probabilities are not known; therefore, all fault probabilities are set to equal probable (1/11) in the algorithm. ** All process faults have random probabilities, and these probabilities are used in the IG algorithm.

**Table 5:** Average number of inspections required and cost for two different scenarios applying a sequential inspection based on IG index and random selection.

	Sequential Inspection with Random Selection	Sequential Inspection with IG Index
First scenario (MMP with high error propagation)	11.5 (1158 €)	5.2 (521 €)
Second scenario (MMP with low error propagation)	5.4 (512 €)	4.7 (445 €)
